# Extracts and Gleanings

**Published:** 1873-06

**Authors:** 


### PART II.

### EXTRACTS AND GLEANINGS FROM OUR EXCHANGES.

### *MULTUM IN PARVO.*

Treating Opacities of the Cornea.—Dr. Charles Bell Taylor, of Nottingham, reports, in the *British Medical Journal*, a case of extensive corneal opacity, in which he tinted away the unsightly defect by the ancient method as reïntroduced and perfected by M. Wecker. He says:

"The operation, which, as a rule, causes very little pain or irritation, is best performed with a number of the finest needles firmly bound with the points on a level around a handle, such as a penholder, or a large needle which has been grooved for the purpose by Messrs. Weiss may be substituted with advantage in certain cases. The substance which M. Wecker recommends for tinting is Indian ink; but I have also employed sepia, ultramarine, and other colors with advantage; and when an immediate and deeply-colored effect has been desirable, a combination of lampblack with Indian ink, and a solution of nitrate of silver. The patient may either recline or be seated in a chair; and it is well to separate the iris with a speculum, and steady the globe with a pair of ordinary forceps, taking a firm grasp of the conjunctiva. The needles are then dipped in one or other of the solutions in question, which should be made as thick as possible, and the superficial layers of the cicatrix are rapidly punctured in an oblique direction, and layers of the solution applied just as in ordinary tattooing, until the white speck is changed from a most apparent deformity into a black surface scarcely visible. A fresh layer of the substance is then applied over the tatooed cicatrix, the patient is directed to keep his eyes open so as to let it dry on and remain as long as possible, and he may at once go about his usual avocations.

"It is important not to close the eye, and to prevent, as far as possible, the washing away of the pigment by the tears. This is best accomplished by enveloping the operator's fingers with a silk handkerchief, so as to mop up the secretion, and afterward by the avoidance of winking on the part of the patient. M. Wecker is content with ten or fifteen punctures a time, and requires from his patients four or five sittings. I have, however, usually completed the operation at once, and make any little addition that might appear necessary some weeks later. The slightest specks, such as are left after phlynctenular conjunctivitis and small ulcers of the cornea, are easily banished by one or two pricks of the needle; and when the whole eye has been opaque, very unsightly, and sight completely abolished, I have very advantageously substituted extirpation of the globe, or the superimposition of an artificial eye, by tattooing a round central pupil so as to restore to a remarkable and most charming degree the natural appearance of the globe. Not only is deformity removed by this slight operation, but patients generally tell us their sight is improved; a fact due, no doubt, to the circumstance that a black speck is much less dazzling than a white one.

"In cases of large cicatrices, I generally make an artificial pupil first, and tint the opacity afterward; and when this is carefully done, it is difficult to distinguish the black speck from the neighboring artificial pupil. I have no doubt that this little operation will also be found of service in cases where considerable dazzling follows the removal of a portion of iris, either *per se* or when the operation has constituted a part of the operation of extraction for cataract; as, by tattooing the cornea, an invisible opacity may be occasioned, which will constitute a permanent shade, and shut out the light to any required extent. Soft cicatrices are readily colored, but old, hard and incrusted ones are more difficult of treatment. In these cases, the nitrate of silver and lampblack are of service; no doubt also lead, sulphur, charcoal, gunpowder, and other ingredients will come to be employed in time. In some cases, the coloration is not very permanent, but it may always be repeated; and even if the perfect blackness do not remain, a grayish, semi-transparent coloration takes place, which looks very like cornea, and it is infinitely preferable to the original deformity.

"It is well to commence the tattoo at the lowest surface to be operated on, in order that the operator's sight may not be obscured by an overflow of the liquid."

ARSENIC IN DISEASES OF THE LIVER.—Dr. de Moura, in the *Gazeta Med. de Balla*, records a case of liver disease, depending on amyloid degeneration of the organ through syphilitic causes, cured by *arsenic*. The patient was cachectic, pale, had polyuria, want of appetite, with a tumefied liver. Vegetable tonics, iodide of potash, iron and mercury had failed to accomplish any good in this case.

Cancer and Carbolic Acid.—Dr. G. M. Pease says: This dread disease has received attention from more charlatans than, perhaps, almost any other, and its treatment is largely in their hands at one stage or another. Many and varied have been the experiments tried, and with hope that it would be found to be the certain mode of cure; but, alas! we have thus far been doomed to disappointment.

That there are cases which are supposed to be cancer, and which yield to treatment by, and give reputation to, quacks, we are all cognizant; yet we equally well know how liable we are to err in our diagnosis, and unless we employ the microscope and actually behold the cancer-cells, we can not be *positive* in our diagnosis. Surgeons of to-day have been greatly jubilant over the drug *cundurango*, from its reputed value for this disease, and yet it proves valueless. What next must we do?

The origin of cancer has been the subject of much study and many theories, the most approved and best received opinion being that it is of germ or parasitic growth. Supposing that to be the case, what should we look for as a means of cure? It is well known that carbolic acid is fatal to all germ life. Why not, then, employ this acid with which to cure cancer?

Acting upon this theory, I have for several years employed it in cases of cancer, with great benefit. It is true I have in every possible case first extirpated the tumor with the knife; there is then less for the system to eliminate, and the cure can consequently be more rapid. In every case thus treated, I have had the best results—none have ever recurred.

The acid is given in two-drop doses, largely diluted with water, twice or three times a day, when the stomach is not empty, but some little time after a meal. This prescription is carried out for three to six months. I have seen cases where diagnosis was nearly certain as to the existence of cancerous disease, which began almost immediately to show signs of recovery, and gradually the tumor would become smaller, and finally disappear altogether. In cancer of the stomach, I have had but one opportunity in which I could have used the acid, and that one was so nearly dead when I first saw her that I did not try it; besides, I had not as full faith in its efficacy as I now have.

The pathogenetic symptoms of a prolonged use of this acid are very like those of a cancer in an advanced stage, particularly of cancer of the stomach. When such symptoms appear to such an extent as to cause inconvenience, it is my custom to either discontinue the use of the acid a short time, or to use it in less doses, or with less frequency. Knowing the tendency to be enthusiastic over a new theory, I have avoided allusion to this subject until my theories could be proven in facts, which, after the lapse of some six years, I think may now be done.

A few cases may serve as examples:

1866.—Mrs. L., age about fifty-five, had a cancer of the mamma, involving the whole gland. The tumor was extirpated with the knife, and the carbolic acid prescribed. The result was a union by the first intention of the wound, and a complete restoration to health. She is still living, and with no indication to return of the disease.

1867.—Miss C., age twenty-two. Cancer of ovary, ovariotomy performed, and carbolic acid administered. Result, complete recovery and no return.

1869.—Miss B., hæmatoid cancer located on the right cheek, extending to ala of nose. Carbolic acid internally and externally, without operation, fully removed all traces after two months' persistence in the use of the acid. Three years have not been sufficient to bring a redevelopment of the disease.

These cases, which exhibit three varieties of the disease, will show the range of action and usefulness of the remedy. Numerous others have been treated, to which the test of time has not been applied, and can consequently only be theorized upon.

If physicians and surgeons will adopt the use of the carbolic acid for cancer patients, they will certainly do no harm, but perhaps may thereby give strength of life and freedom from pain to their suffering dependants.

Remedy for Tapeworm.—Prof. J. F. Locke (*Eclectic Medical Journal*) writes: Dr. —— called on me, saying that he was troubled with a tapeworm, parts of which had escaped or had been expelled on different occasions. He had taken koosso, male fern, and at different times other remedies in greater or less repute. I assured him that I could secure the expulsion of the worm, and volunteered to prepare and administer the remedy. I took of the bark of the pomegranate root, *half a pound;* to this I added two pints and a half of water, and boiled the liquid down to one pint. Of this decoction I gave the patient six ounces, after adding five drops of the oil of anise, and a drachm of the fluid extract of jalap. I should have given about the same amount in two hours if the desired effect had not been produced. In forty minutes after the first and only dose was swallowed, an evacuation of tbe bowels occurred, and the entire worm—head and all—was found to be expelled. In about fifteen minutes after the medicine had been taken, nauseous sensations were excited which resulted in a slight attempt at vomiting, but not enough fluid was thrown from the stomach to interfere with an efficient action of the medicine downwards. The patient sucked a lemon to allay the nausea.

To complete the report, I will say that six hours before the remedy was given, an active cathartic was taken, with the idea of preparing for a speedy contact of the pomegranate with the worm.

In this case the effect was rapid and satisfactory, no part of the parasite being lost. In some cases the slender neck of the worm might be severed in the act of expulsion, and pass unobserved, though the death of the creature might be assured. The presence of the entire worm in the discharge is alone satisfactory. If the head of the parasite does not appear in the evacuation, it is best to administer a second dose of the medicine, and await its action. If, at a subsequent period, it be known that the worm was being reproduced from the unexpelled and living head, a repeated trial of the medicine might be made, but I have not had a failure. If I ever fail with the dose recommended, I shall administer a larger one.

It is presumed that the worm inhabits the small intestines, but if its habitat happened to be lower down, a greater quantity of medicine might be necessary to reach it. The nearer the parasite is to the stomach, the more hastily does the medicine impress it, though the proof of death in the worm is in the dislodgement. The length of time demanded for expulsion depends upon the cathartic effect of the remedy.

In conclusion, I deem it of consequence to say that the decoction should be prepared in an earthenware vessel, and that the pomegranate bark should be of good quality. Bark a year old, if properly preserved, retains its qualities in sufficient strength to prove efficient. Care should be exercised to guard against adulterations.

To avoid misapprehension, and to place the prescription and method of preparation in a compact form, I will repeat to some extent what has already been said: ℞. Take a half pound of bark from the pomegranate root; add two and a half pints of boiling water; let the mixture stand in a warm place for at least two hours; then boil down to one pint; strain while hot through a fine wire strainer. To every six ounces of the decoction, add one drachm of the fluid of jalap, and five drops of the oil of anise. Six ounces of the prepāration is regarded as a dose; and the medicine should be given warm. It should be repeated every two hours until the worm is expelled. Three hours prior to the administration of the first dose, the bowels should be evacuated by the action of a cathartic, none being better than our anti-bilious physic.

PROF. PARKER'S TREATMENT IN THE TERTIARY FORM OF SYPHILIS.—℞. Iod. patass., ℈iv.; ext. conii, gr. xvj.; aquæ, ℥iv. M. Dose, a tablespoonful twice a day; after a time, it may be taken three times a day.

The Treatment of Goitre.—This topic is treated of by Dr. C. Schwalbe, in the last volume of *Virchow's Archiv.* His article is interesting, the results arrived at being based on one hundred and six cases, of which notes were made. In order to the treatment of goitre, it is of consequence to decide as to the exact nature of the swelling, and so the author gives a short description of the various kinds, chiefly with the view to diagnosis. There is first the glandular form, (struma follicularis mollis of Virchow), which consists of a hypertrophy or hyperplesia of the thyroid gland, and which forms a soft and elastic tumor, not unlike a lipoma. There is, further, the cystic goitre, in which it is usually easy to detect fluctuation. The commonest remaining forms are the fibrous and vascular, the former distinguished by its hardness. As an important aid to the diagnosis, the author nearly always resorts to puncture with a perforated needle, and the proceeding is of particular advantage in distinguishing between glandular tumors and cysts. The glandular tumors not unfrequently give a false feeling of fluctuation; and on the other hand, it is often impossible to detect fluctuation though the tumor is cystic, and in these circumstances puncture is of great service; the fluid which issues when the disease is cystic at once determines its nature. The fibrous forms are also distinguished on puncture by the density of the tissue, and the vascular by the free flow of blood. In his treatment, the author trusts, in the first place, to the internal administration of iodide of potassium, combined in almost every case with the local injection of tincture of iodine, or simple alcohol. In very recent cases the use of the iodide (he gives it to the amount of one gramme, or about fifteen and a half grains daily) is sufficient to dissipate the swelling, but usually injection is also necessary, except in the vascular forms.

In respect to the material to be used in injection, the author believes that the benefit derived from the injection of tincture of iodine depends chiefly on its alcohol, and that rectified spirit is, in most cases, of equal use. The internal use of KI or I is undoubtedly of great benefit, but there is no reason to believe that the iodine has any special effect when applied locally, and the author has found that the injection of alcohol with the internal administration of the iodide has as good results as the injection of the tincture of iodine. To the surgeon, there can be no comparison of the cleanliness and convenience of alcohol as compared with the tincture of iodine.

The quantity injected varies in different cases. In the glandular forms, only small injections were used—about ten to twenty drops; and even smaller quantities in the fibrous. But in the cystic form, much larger quantities were used, as much as four grains being injected into a cyst as large as a duck's egg. The author has not made much of electrolysis in the treatment of goitre; and he employs it chiefly for the vascular and fibrous forms. Incision of the cystic form he has very seldom resorted to, and only where the tumor was very large.

In conclusion, it is stated that by these means he has only once failed to effect a cure, this case being one of the very hard, fibrous form. At the time it was under treatment, he had no constant current battery at hand, and he thinks it possible that he might have come to some result if that treatment had been used.—*Medical and Surgical Reporter.*

TRANSMISSION OF SYPHILIS.—Dr. John S. Parry, in a recent clinical lecture at the Philadelphia Hospital, (*Philadelphia Medical Times*, September 16, 1872), inculcates the following sound doctrine with regard to the propriety of marriage of syphilitics:

"I have for some time availed myself of every opportunity to try to obtain information upon the relative liability of each parent to transmit the disease, and, while I may subsequently have to modify my views in regard to it, I cannot but believe that the mother is much more likely than the father to transmit syphilis to the children, and that, as Mr. Morgan says, the influence of the father has been exaggerated. On the other hand, I do not want you to understand me as saying, with M. Cullerier, that syphilis can not be inherited from the father. I can not at present commit myself to any such doctrine, for I have seen more than one sad instance in which men *seem* to have begotten syphilitic children.

It is important that you should inform yourselves thoroughly upon this subject, for gentlemen who have been relieved of secondary symptoms will hereafter consult you in regard to the propriety of marriage. Every physician with any experience can point to numerous examples of this kind. It becomes you, therefore, to be able to give a reliable opinion in regard to it; and I beg you, I beseech you, gentlemen, to remember that when a man has once had constitutional syphilis, you can not, in the present state of our knowledge, say positively that he will not procreate a diseased child, no matter if he seems to have fully recovered. You may feel assured that he probably will not, and more, you may be certain that the probabilities are reduced to a minimum; but, as we now understand these matters, you are not justified in giving a positive opinion in regard to this subject. If a man has once had constitutional syphilis, we do not doubt that, under certain circumstances, the poison may be eradicated so far as to prevent his begetting a diseased child; but we have as yet no reliable means of judging when this is the case.

If your opinion be asked upon this important question, it is your duty to give it clearly and truthfully, without regard to the feelings of your patient. It is your duty to yourselves, to your interrogator and to his prospective children. But when you have told him all this, you have only performed a portion of your work. You have seen only one side of the dark picture. This is a cloud which has no silver lining. Not only is a man who is affected with constitutional syphilis to be told that he may transmit the disease to his children, but he is likewise to be informed that his wife may contract it from him, or from the child in her womb, and that he may in the future have to bear the terrible trial of seeing her suffer from secondary and tertiary syphilis.

I know that there is not one of you who will not shrink from such a trying position as this. With a human being before you in whose heart burn the same passions, the same loves and hates that move you, you will feel tempted to put the case in the most favorable light. But, gentlemen, tell the simple truth, remembering the delicacy of your position and the dignity of your calling—remembering that in your decision are involved the life and comfort of unborn children, the health and happiness of a woman who is about to give her heart's best treasures into another's keeping.—*Northwestern Medical and Surgical Journal.*

On the Pulvis Glycyrrhizæ Compositus, a Laxative Preparation of the Prussian Pharmacopœia.—David Page, M.B., of Kirby-Lonsdale, England, says:

"For the treatment of simple constipation resulting from atony of the bowel, the compound liquorice powder is admirably adapted. Whether in simple uncomplicated torpor of the intestines, or in constipation accompanying temporary gastric disorder, the powder, alone or as an auxiliary to appropriate remedies, is preferable to other preparations of its class. In the former, our object is rather to call into play the peristaltic action of the intestine, than to deplete by serous transudation from its walls, and, in the latter especially, no prudent practitioner would run the risk of aggravating the disordered stomach by the exhibition of purgatives possessed of irritant or drastic properties. The compound liquorice powder is composed of the following constituents, so prepared as to form when incorporated an almost impalpable powder:

"℞. Senna leaves, ℥ vj.; liquorice root, ℥ vj.; fennel seeds, ℥ iij.; sulphur, ℥ iij.; refined sugar, ℥ xviij. This formula is given in the Pharmacopœia Borussica.

"The active ingredients are sulphur and senna. The action of the former, when administered alone, is frequently accompanied by tormina, and its continued use is apt to cause derangement of the mucous membrane of the upper intestine. The physiological action of sulphur appears to be upon the muscular coat, and less upon the mucous surface, while senna is a more active purgative, more apt to excite tormina, and acts more upon the mucous than the muscular coat. By the aromatic and stimulant properties of the fennel, and the demulcent action of the liquorice, itself a mild laxative, the effects of the more active constituents are judiciously modifiad.

"The usual dose is a small teaspoonful at bedtime in water, with which it is easily mixable, forming an agreeable draught. Children, to whom Gregory's powder is a terror, readily take it with a belief that it is a sweet-meat.

"That the action of the powder is not to produce catharsis with serous transudation is proved by the motions, which are usually well formed and soft."

Hypodermic Use of Varatrum in Meningitis.—Dr. L. J. Collins (*Clinic*) writes:

"January 2, 1873, I was called to see James B——, aged eight years, of slender stature and strumous habit. I elicited from the parents the following history: The boy had been complaining of headache for several days previous. On the morning of January 1, he was seized with violent pain in the head, seated principally in the frontal region; vomiting set in about the same time, and continued almost incessantly until I saw him.

"I found him in a semi-conscious condition, head slightly retracted, tongue coated with a heavy whitish fur, pulse 134, temperature 104½°, respiration 40. When aroused, he complained of pain in the head. Pupils contracted to the size of the head of a pin, with convergent strabismus, stronger marked in the left eye. Considerable muscular rigidity, especially marked in extremities, the finger-tips being drawn almost to the wrist. Intense thirst, water rejected as soon as swallowed.

"After several ineffectual attempts to administer medicines by the stomach, I gave an enema of castor oil and molasses, which brought away a small quantity of fecal matter. I also administered gtt. iij. veratrum subcutaneously. In one-half hour the characteristic impression of the veratrum was noticed on the pulse. One-half hour later the pulse stood 108, temperature 103°. Gave another drop of veratrum hypodermically; applied cold compresses to the forehead. One hour later (two hours from the first injection) the pulse was 82, temperature 101°, respiration 25. Opisthotonos disappearing; stomach becoming tolerant. I now gave calomel grs. x. with croton oil gtt. j. made into a bolus with butter; applied a fly blister to the nape of the neck extending from the occiput to the sixth cervical vertebra; continued the veratrum in gtt. j. doses by the stomach. Eight hours after my first injection, pulse 62, temperature 99½°, respiration 22, no opisthotonos; pupils almost normal in size, photophobia much diminished. Ordered potass. brom. grs. x. every three hours, with chloral hydrate grs. x. to be given at night.

*January* 3.—Patient perfectly conscious, but very weak. Continued the potass. brom., with quin. sulph. gr. ss. every three hours. Liquid nourishment.

*January* 4.—Patient better in every respect. Strabismus still perceptible in the left eye.

*January* 5.—Patient discharged, and has contained well up to the present writing, January 27.

Taking into consideration the fact that he was of a strumous habit, having once been treated for scrofula, I was not prepared for the result of my treatment.

Fibroid Tumors of the Uterus.—Alfred Meadows, M.D., London, England, (*American Journal of Obstetrics*), in his "Remarks on the Diagnosis and Surgical Treatment of Fibroid Tumors of the Uterus," says that, having determined the situation of the tumor and its interstitial character, one is justified in attempting the removal of these tumors, even though they be not intra-uterine or submucous, but are situated in the substance of the uterus itself, provided a proper canal be inaugurated. His plan is, first of all, to prepare the passages for the expulsion of the growth; and, secondly, to detach the tumor from as much of its surroundings as possible; so that, by making of it a foreign body, nature may aid in its removal, as she would in the case of a dead fœtus or a mole-pregnancy, or even a uterine polypus. Lastly, when nature has been given fair play, the ecraseur should come to the rescue, and remove at once what might otherwise be the work of many months or years. He had recently under his care a case in which the tumor was completely imbedded in the substance of the uterus, so much so that the os was not dilated in the very least; and he had the satisfaction, after three or four operations, of completely removiug the tumor, which was of the size of a small cocoa nut. The patient is now perfectly well.

At the date of writing, he had two other cases of the same kind, but in both the tumor was much larger. He had commenced with the same plan of treatment in these cases, and he had every reason to believe that a cure would be effected.

The first step in the process is to prepare the passages for the removal of the tumor. For this purpose he recommends free division of the cervix uteri in one or more directions. The next step is breaking with the finger through the capsule, and little by little detaching the tumor from its bed. During the intervals, efforts should be made, by the administration of ergot, borax, cinnamon, and other so-called oxytoxics, to secure contraction of the uterus, so as to favor nature's method of expulsion. Galvanism is also another agent of great power in this respect, and a firm bandage is of service in cases where the tumor is large and projects well into the abdominal cavity.—*Medical Record.*

Catarrh of the Nose.—Dr. James A. Fitzsimmons (*Chicago Medical Examiner*) employed the following treatment in a case of eight years' standing:

"The patient was placed on a chair, with a spittoon directly in front of him. I prepared a solution of nitrate of silver, grs. xxx. to the ounce of water; taking a common catarrhal syringe, I filled it about three parts full; as much as three drachms of this was thrown up through the posterior nares; the instrument was quickly removed, and the patient instructed to throw his head forward and downward, so as to permit the fluid to pass through and escape out of the nose. Where there is no obstruction, this will always occur, provided the patient be always instructed as follows: After being comfortably seated, he should be instructed to open his mouth, fill up the lungs, and hold his breath. The operator must now at once introduce the syringe into the mouth and throat, pass it at once behind the palate, push it upward as far as the bend (as much as an inch), and throw the contents in as quickly as possible, never giving the patient time to draw his head away. In taking out the syringe, the palate should not be pulled nor drawn outward by the bulb on the point of the syringe. *Elevate the handle, turn the point downward at the same time*, and no injury can result. The application in this instance caused a good deal of pain, stermitation and lachrymation, which continued one or two hours. Immediately after the injection I used a probang, saturated in the same solution, and freely touched every part of the throat, and also passed it for some distance into the œsophagus. Following these applications there was a great quantity of ropy, whitish mucus discharged by blowing and hawking; the spittoon was, I should think, half filled with this tough mucus; when poured out, the last of it drew out into a long, stringy mass.

"The patient continued to suffer a good deal of pain in his head during the night, and vomited a little he had eaten. The following morning he reported himself as feeling considerably better, had partaken of a little more food than was usual for him, and felt less pain in the stomach. On the second day I used ten grains of nitrate of silver to one ounce of water, and again used the probang, and prescribed the following mixture for the stomach: ℞. Nitro muriat. acid, ʒ ii.; tinct. gentian, ℥ i.; infu. gentian, ℥ iv.; tinct. card c., ℥ ss. One teaspoonful of which was ordered to be taken before each meal. The medicine was not procured until the following day, but on the third morning he reported himself as feeling decidedly better; had slept soundly, had taken considerably more food, and felt little pain. I now substituted carbolic acid and glycerin in place of nitrate of silver, and used these with a hand atomizer, showering the throat and nasal cavities regularly once a day. When there were indications present for the use of nitrate of silver, this was used, after the parts were cleansed thoroughly with the douche and showers of spray.

"These measures were persevered in for about six weeks, at the end of which time my patient was entirely well, could eat any thing he wanted, and had gained thirty pounds of solid flesh. It is now more than a year, and he has continued to enjoy uninterrupted good health; occasionally yet he has some discharge from his throat after exposure to cold."

Treatment of Whitlow.—Dr. J. B. Mattison (*Medical and Surgical Reporter*) writes:

"Early incision is the one above all other remedial measures, on the urgent necessity for which we desire to lay particular stress. But when and where are we to make it? Pus gives early indication of its presence by throbbing, pulsating pain. The moment, therefore, such an event declares itself, if not before, surgical assistance should be sought, and the affected part thoroughly incised. Thoroughly, we say, for no half-way measures are admissible under such circumstances. The knife must be handled boldly, and the pent-up pus allowed free exit, if we would effectually relieve suffering, save structure, and conduce to a speedy, favorable termination.

"We think that among the unprofessional there is a lack of correct information on this point which physicians could and should supply. Common enough, too much so, in fact, it is to see patients suffering for days the agonizing pain of a felon, and yet absolutely refusing to submit to the knife under the ridiculous plea that it is 'not ripe.' Now, this is altogether wrong—wrong, in a measure, on the part of the physician, in allowing his patients to entertain such an opinion, unless he has utterly failed in a determined effort to disabuse their minds of such an erroneous idea; and entirely wrong on the part of the patient. Practitioners ought, in each and every instance of whitlow which comes under their observation, to forcibly impress upon the mind of their patients the transcendent importance of this procedure, and instruct them carefully upon the earliest indication for its employment. It is only by such action on the part of physicians that this wide-spread error can be corrected, and an immense amount of suffering, which would otherwise necessarily ensue, be averted.

"But where is the incision to be made? Huetter answers this question. According to Dr. Proegler, he takes a fine probe and touches the central part of the panaritial swelling, asks the patient where he feels by this first palpation the most intense pain, and makes there the incision. This point covers in the beginning of the panaritum not more than a quarter of a line, but if taken for the incision no one will be misled.

"Huetter asserts, by following this plan, he never made a useless incision. This is a most important statement, and if his success extends to the same operation in the hands of other surgeons, it becomes an infallible guide, and one of which we can by no means refuse to avail ourselves."

Veratrum Viridi.—Dr. Eugene Peugnet (*Medical Times*) remarks:

"Veratrum viride is not so much in vogue as formerly, owing to the uncertainty of its preparations, to its misapplication, and to its disagreeable and even somewhat alarming effects.

"The objection to the uncertainty of its action is easily guarded against.

"The one against its misapplication lies in the power of every practitioner to obviate; that is, accuracy of diagnosis, a proper appreciation of the therapeutical applications, and a proper knowledge of its physiological action.

"As to its disagreeable and alarming effects. The first are easily guarded against, by giving a few drops of Magendie's solution with it, which is rarely necessary after the first dose. For children, a few drops of paregoric will answer. In reference to the latter, they have been, I am inclined to believe, greatly exaggerated, and they are in a great measure due to the manner in which it has been generally administered—that is, five to six-drop doses hourly, and increased *guttatim*. Although the remedy is not cumulative in its sedative action on the pneumogastric nerve, and through this action it causes nausea and vomiting; but if we administer a full dose—ten to fifteen drops—we have at once a decided sedative action, and we can regulate it by a proportionate reduction of the dose, and a proportionate interval between each one. Whilst I have seen unpleasant effects induced by increasing the dose *guttatim*, I have never seen any unpleasant effect from adopting the opposite course.

"I know of no agent in pharmacy which is more potent and decided in its action as an arterial sedative and a depressant of caloric. It acts much more rapidly than aconite; moreover, its action is entirely different. Aconite acts primarily on the spinal centres, and its secondary action on the vaso-motor system is two-fold; primarily, in small doses it acts on the cardiac plexus, causing a contraction of the vessels, diminishing the flow of the blood; secondarily, in three to five-drop doses it causes a dilatation of the blood vessels, and the rapidity of the circulation is greater. Hence its therapeutical application under the accepted view of the phenomena of inflammation would be due to its primary action and in small doses; but I must confess that I have never been able to see its decided action on the circulation in the more acute inflammatory affections, and in the dynamic fevers its use is certainly contraindicated; whilst if the accepted view of the phenomena of inflammation is correct, we should endeavor to reduce the increased action of the circulation, and to diminish the temperature, which can only be done by paralyzing the vaso-motor nerves, and that action appertains *a fortiori* to veratrum.

"The action of aconite is *par excellence* in febrile reactions with *peripheral nerve pains*, in neuralgia involving the peripheral nerve expansions, upon the local and dermal hyperæmia due to an arthritic diathesis; thus demonstrating its special and primary action on the spinal nerve-centres, by diminishing the hyperæsthesia."

Removal of Portions of Diseased Tissues in Cases of Carcinoma Uteri.—Dr. Alexander Simpson, Professor of Midwifery in Edinburgh, gives his experience in this procedure, as follows:

"Agreeing with most gynæcologists in the propriety of amputating the cervix when by so doing all the diseased tissues can be removed, he thinks that benefit may be derived by cases more hopeless, by removing as much as possible of the cancerous growth. Regarding the dangers of operative interference arising from hemorrhage, pain, aggravation of the disease and risk of life, he thinks: 1. Although digital examination of a case of carcinoma uteri is apt to be attended with a certain amount of hemorrhage, the bleeding is all from the surface, and in the case of removal, the bleeding lessens as healthier tissues are reached. 2. As for the pain produced, it will be found, when anæsthesia is not resorted to, that though these patients suffer commonly enough to a degree peculiar to their disease, yet operations for the removal of the diseased mass are not attended with much suffering, and may be performed without the aid of an anæsthetic. 3. It need not be apprehended that the progress of the disease will be hastened by this kind of interference; for experiments show that the more thoroughly the disease is removed, the longer it is in returning, and is no more rapid in its course, when it does return, than when left to itself. 4. The operation is not dangerous to life. Out of twelve cases operated upon by himself, and a large number similarly treated by Sir Jas. Y. Simpson, only one case resulted fatally, and in that death was due to collapse.

"The advantages to be derived from the operation are: 1. Arrest of hemorrhage, the persistence of which exhausts the patient. In many cases the bleeding takes place in part, no doubt, from the mucous membrane, which becomes hypertrophied as a result of the excitement in the whole organ attendant on the presence of a morbid growth; but whether the source of the bleeding be menorrhagic or metorrhagic, it is always lessened, and sometimes arrested by the removal of accessible portions of cancerous tissue. This is markedly the case when we have to do with the so-called cauliflower excrescence, or with an expanded epitheliomatous os. 2. And nearly as important is the arrest or lessening of the fetid discharge, which of itself renders the patient miserable. 3. In a case of cancer of the cervix uteri, pain is not necessarily a prominent symptom, until the disease has made great progress, being sometimes quite insignificant; but where the disease involves the uterine cavity, pain is an early, most distressing, and almost constant symptom, and the relief afforded by ablation of even a portion only of the diseased tissue is immediate and striking. 4. Although but little can be said of the effect of the operation in prolonging life, owing to the limited number of cases which have been treated, Dr. Simpson has seen no instance in which life appears to have been shortened, and he is of the belief that the arrest of the exhausting discharges and the relief of pain must of themselves tend to lengthen the patient's existence.

"Not only are cases of cauliflower excrescence, or mushroom-like epithelioma of the os uteri where the cervix is healthy, suitable cases for this mode of treatment, but so, also, are cases of encephaloid character, with projecting, hyperæmic masses; schirrus, and other varieties, which present an excavated surface, and when ulceration has not perforated the uterine and vaginal walls.

"The galvano-cautery, the *écraseur*, and the *curette*, are the instruments recommended."—*British Medical Journal.*

The Treatment of Sciatica.—David Pride, M.D., of Neilston, writes to the Glasgow *Medical Journal:*

"The intractable nature of this disease, and the great amount of suffering it entails—at times keeping even the most powerful man completely under its thrall for weeks—renders any mode of treatment which has been followed with good results worthy of being recorded.

"C. D., aged forty years, a strong, healthy, well-made man, gave his body a sudden jerk, by trying to throw a parcel of goods up to a person in the flat above him. He instantly complained of severe pain in the gluteal region, which extended down the back to the thigh, in the course of the sciatic nerve, to the lower leg, and he had to be taken home in a cab. I saw him afterwards, and at different times prescribed sinapisms and rubifacient liniments externally, and the iodide and bicarbonate of potash, iron, arsenic, etc., internally, but with very little benefit. At length the patient was put under chloroform, and the actual cautery applied over the course of the nerve, confining its application to the posterior aspect of the thigh; and this was repeated in the course of a day or two, with the happiest results. Patient got rapidly well, and, after walking about somewhat lame for a few days, ceased to be troubled with the affection, and has had no return of it. There can be little doubt but that in this case the affection was due to rupture and consequent inflammation of some of the component fibres, and their sheath, of the sciatic trunk.

"J. L., aged fifty-five years, a miner, has for years been employed in damp and wet underground workings, but never before had any thing the matter with him like the present affection. Complains of severe pain coursing down the back of the thigh to the outer aspect of the lower leg, which quite screwed him up, and prevented him from working. Cupping, sinapisms and liniments were tried externally, and iron, iodide and bicarbonate of potash, colchicum, Fowler's and Donovan's solution, at different times internally, but with very little benefit; at length the actual cautery was used in the course of the great sciatic nerve in the thigh and hip; the result was every thing that could be desired. He gradually but completely recovered, got the use of his limb, and has had no return of the disease since.

"R. S., aged forty-eight years, a miner, complained of severe pain in the back of thigh, extending to the outer ankle, but most severe in the calf of his leg. In this case, also, the alkaline and arsenical preparations were had recourse to, and with this benefit, that the pain got confined to the calf, but here it continued very persistent. In this case I used the hypodermic injection of the liq. opii sed. with most marked benefit after two or three applications. Patient rapidly recovered, and was able to return to his work. Has been quite free of the disease since.

"This treatment by the actual cautery merits a more extended trial. It will be found especially useful in cases where the affection is due not so much to any rheumatic element in the system, as to local injury to the component fibres and funicular sheaths of the nerve itself, as in the case of C. D."

An Improved Method of Taxis in Strangulated Hernia. Dr. F. H. Nichols, Cumming, Georgia, (*Medical and Surgical Reporter*), remarks:

"If there is great tenderness over the hernia, I apply a warm, soothing poultice, with or without hot cloths saturated with chloroform, over the immediate stricture for half an hour, while I am getting other things ready. I place the patient upon his back and verging on the side of the hernia, with his lower extremities drawn partly up so as to relax the muscles in front of the abdomen. I engage his attention with cheerful conversation, and assure him of his speedy relief, which I know I can certainly give him. I have placed at the side of the bed a bucket of cold water, the colder the better. Having all things in readiness, I gently raise up and compress the hernia with both hands, holding it so that the pressure will be in the line of the passage constricted. I place the shoulders a little lower than the hips, with one knee generally flexed a little. I faithfully but gently work back the protruded part, and in many instances succeed in reducing the strangulated bowel and restoring it into the belly.

"But there are cases that *taxis*, however skillfully performed, can not reduce the hernia. In such cases, I do not despair. I carefully readjust the pressure, reduce or lessen the tumor as much as possible, holding the part and making the pressure close to the point of stricture with one hand, and with the other, after holding it in cold water a few minutes, *I suddenly seize the abdomen below the navel and carry it upwards*, at the same time using a little more force or pressure with my other hand at the stricture. The shock of the cold wet hand, and the continued gentle pressure at the stricture, never fail to unlock the bowel, while the patient cries out, Oh! only to find himself completely relieved, and the hernia gone—gone into the abdomen, and the cure is completed. I thus excite the retractive power of the bowels as well as of the whole abdomen. This power is adequate for the reduction of many cases of strangulated hernia alone. It becomes potential and all-sufficient when conjoined with proper pressure at the point of stricture; and amply so for every case of strangulated hernia that I have ever seen. *I know this by experience, and I fully believe that it is the remedy "par excellence" in every obstinate case of strangulated hernia.* This retractive power can be excited at our will, can be united with external pressure, and is without risk to the patient, and always in my hands it has proved successful. I invite all medical men to try it for themselves."

Freckles, it is said, are removed by the application, twice a day, of powdered nitre moistened with water.

Aural Affections in Children.—Dr. Böke, in the *Jahrbuch für Kinderkrankheiten,* remarks that aural affections of children are most frequent between the ages of three and seven, and this is due to the circumstance that acute exanthematous affections, which are associated with affections of the tonsils and of the mucous membrane of the throat, are then most common. Such affections are apt to propagate themselves along the Eustachian tubes into the tympanitic cavity, the lining membrane of which, as above stated, presents all the characters of a mucous membrane. Hence, also, the most common form of disease in childhood is otitis media. Primary disease of the external auditory meatus is more frequent before the age of seven than afterward, when it is apt to accompany or be secondary to disease of the middle ear, rendering it difficult to determine which was first affected. Dr. Böke refers otitis externa to carious teeth, since the external auditory meatus and the jaw are both supplied by the same nerves and vessels. For the treatment of discharge from the ear he recommends injections of warm water and insertion of a plug of cotton wool. Five drops of a lukewarm solution, containing two grains of acetate of lead in two ounces of a mixture of water and glycerin, may after each injection be dropped into the ear. The diseases of the middle ear are divided by the author into inflammation and catarrh. Inflammation in the tympanum is accompanied by very violent symptoms, fever and cerebral excitement, and leads to perforation of the membrana tympani and purulent discharge. If the discharge have only lasted a few days, it may be injected two or three times with lukewarm water; but if it have lasted longer, astringent solutions should be dropped into the ear. Polypi are to be touched with nitrate of silver which has been melted in a porcelain capsule and allowed to concrete to the size of a mustard-seed on the end of a probe. Treatment is required for the space of six weeks; perforations of the membrana tympani heal. Complete deaf-mutism developing in children is usually the consequence of brain disease.

On Temperature in Phthisis.—In an inaugural thesis published on the above subject by Dr. Bilhaut, the author puts down the following, among other conclusions: From the outset of the disease the temperature rises; toward the end of the disease there is often a marked decrease of temperature; diarrhœa and abundant hemoptysis produce a fall of temperature; differences of temperature show the gravity of the disease; slow asphyxia and inanition induce a fall of temperature toward the approach of death; the tracings in cheesy pneumonia seem to be more regular as regards vesperal exacerbations and morning remissions than the tracings in tuberculosis; the complications of the disease modify the form of the tracings.

Treatment of Erythema.—Dr. H. S. Purdon, of Belfast, Ireland, in a short paper on this subject, in the Dublin *Journal of Medical Science*, says that, as a rule, he finds no difficulty in managing erythema nodosum by prescribing the compound iron mixture and compound decoction of aloes combined, and adding, in cases where the catamenia is suppressed or scanty, to the mixture borax; while locally, if pain is much complained of, a lotion of acetate of lead and opium or iodoform ointment (one drachm of iodoform to one of lard, a few drops of rectified spirits being used to dissolve the iodoform), which is a good anesthetic, are used. A flannel bandage being applied every morning and worn during the day, however, especially in people more advanced in years than those to which my remarks refer, and in whom this disease is not only rare, but also very obstinate, the peroxide of hydrogen and tincture of the perchloride of iron are the remedies to be recommended. The dose is a teaspoonful of the peroxide and ten to twenty drops of the tincture of iron in a wine-glassful of water thrice daily. (It will not do to order these two medicines together as in a mixture). Dr. Brown-Sequard has recorded the fact that oxygen and strychnine contained in the blood have the power of acting on the excitability of nerve fiber in the nervous centers or in nerve trunks of filaments, and thus the peroxide, he thinks, in erythema nodosum tends materially toward restoring a healthy tone in the part affected. If the premonitory symptoms of fever with which the eruption is ushered in be at all severe, rest in bed and an effervescing saline aperient, is all he considers that is necessary.—*American Practitioner.*

Chloroform in Labor.—Harvey L. Byrd, M.D., of Baltimore (*Physician and Surgeon*), gives the following observations concerning the use of chloroform in labor. It is of the greatest moment that the pulse and respiration be carefully watched from the commencement to the close of its administration, as much so, in fact, as in any other condition of the organism justifying or calling for its administration, if danger is to be avoided from its use. These functions afford the only accurate and sure indications of the propriety or impropriety of continuing the inhalation. The administrator should stop immediately, if the pulse becomes weak and the respiration difficult or irregular. Any hesitation or faltering in either should demand the instant cessation of the inhalation. Careful attention to these facts will enable physicians to keep the larger majority of women sufficiently under its influence, when desirable to do so, for hours; and thus, through its agency, labor may, when necessary, be rendered absolutely painless.—*New York Medical Record.*

Baptisia Tinctoria in Typhoid Fever.—Edward Duffield, M.D., in the *Medical Record*, gives the history of two bad cases of typhoid fever, where, after trying nitric and sulphurous acids with quinine and extract of belladonna, and the turpentine emulsion, and, failing to relieve, or even make any decided impression on the disease, he at last resorted to the baptisia tinctoria, or wild indigo, with decided success. In his own words: "We gave the concentrated tincture in three-drop doses every two hours, continuing the acid and belladonna; and with what torturing anxiety we waited and watched for hours, to discover any effect it might have, can be divined only by those under like circumstances. Finding it borne well, after six or eight doses had been tried, it was increased to five drops with the same interval of administration; and at the expiration of twenty-four hours we could discern a reduction of a degree or more in the temperature, with softer pulse and skin, and so felt encouraged to persevere. At the expiration of forty-eight hours there was less delirium and less subsultus, etc. In seventy-two hours from the commencement of it, there was some gleam of intelligence, tongue softening and sordes diminishing; and, at the expiration of another twenty-four hours, there was a return of consciousness sufficient to recognize the calls of nature by the patients, and the direction of the nurses' attention to them. Diarrhœa very much diminished, and their beef tea, etc., taken readily. The treatment was continued a week, when convalescence had established itself. The effect of baptisia was so manifest in these two cases as to induce me to try it in some eight other cases of typhoid fever, the last one of which is now just convalescing. Whilst we do not desire to be over-sanguine, and are frank to admit that its trial in ten or eleven cases is not sufficient to establish its full value; yet, it is sufficient to assure us of its power thus far, and to ask that the medical profession shall give it a full and fair trial for themselves."

A physician of this State (Michigan) has informed the compiler that during a severe epidemic of typhoid fever on his ride, he used the baptisia tinctoria with the most marked success. His method of administering it was practically the same as that of Dr. Duffield. *Detroit Review of Medicine.*

Phytolacca in Mastitis.—Dr. G. W. Biggers writes from La Grande, Oregon, to the *American Medical Journal*, that he has never failed in curing mammary inflammation by giving phytolacca decandra, in the form of fluid extract, fifteen or twenty drops every three hours. He has used it repeatedly, and the cure is accomplished in thirty-six or forty-eight hours. The lochia, when suppressed, are restored by it.

Local Uses of Tannin.—Dr. G. P. Hachenberg (*New York Medical Record*) reports several cases of the use of this remedy in prolapsus uteri, where other means had failed to afford relief. His method is as follows: A glass speculum is introduced into the vagina so as to push the uterus into its place. Through the speculum a metallic tube or syringe, with the end containing about thirty grains of tannin, is passed. With a piston the tannin is pushed against the uterus, the syringe withdrawn, and the packing neatly and effectually completed with a dry probang, around the mouth and neck of the womb. After the packing is completed, the probang is placed against the tannin, in order to hold it, and the speculum is partially withdrawn. The packing is now fully secured, and the instrument removed.

The application of tannin holds the uterus firmly and securely in place, not by dilatation of the walls of the vagina, but by corrugating and contracting its parts. At first the application may be made weekly; finally, but once or twice a month. It not only overcomes the hypertrophy and elongation of the cervix, but even, the writer thinks, induces a slight atrophy of the parts. As a remedy for leucorrhœa, where the seat of the inflammation is at the mouth of the womb, or within the vagina, it actually gives speedy relief. The doctor also reports a case of chronic ulceration of the rectum which was cured after a few weekly packings of tannin. He has found, moreover, that in affections of the throat, direct applications of tannin to the diseased parts gives satisfactiory results. In a case of extraordinary hypertrophy of the tonsils, preparatory to the operation of extirpation, tannin mixed with tincture of iodine to the consistency of syrup, was applied with the effect of so diminishing the hypertrophy that a surgical operation will, in all probability, not be necessary.

No remedy has given such satisfactory results in certain forms of chronic ophthalmia and opacity of the cornea, as tannin once a week placed under the eyelids—pure, well trituraied tannin. An aged lady, who had chronic ophthalmia, was relieved by one application; another, who was blind from opacity of the cornea and chronic ophthalmia, recovered her sight mainly from the local use of powdered tannin.—*Boston Medical and Surgical Journal.*

Purulent Ophthalmia Treated by Alcohol.—M. Lannelongue, in the *France Medicale,* recommends the use of alcohol in purulent ophthalmia. The usual strength is one-third alcohol to two-thirds water. He thinks the injections act both by their direct effect and as a wash, and have been very successful in his experience. The proportion of alcohol may be increased or diminished as the cases seem to demand.

Local Pain in the Right Side in Pregnancy.—This symptom, occasionally seen in obstetrical practice, is the subject of an inquiry in the *British Medical Journal.* Some of the replies are as follows:

"The local pain in the hypochondrium, mentioned as occurring in the latter months of pregnancy, is probably due to irritation of and pressure on the hepatic nerves, which are chiefly derived from the solar plexus. Having had several similar cases lately, which have derived marked benefit from the following formula, I would recommend him to make trial of it, and shall be glad to know the result: ℞. Potassii bromidi, ʒj.; tinct. hyoscyami, ℥ss.; spiritus chloroformi, ʒj.; aquæ ad, ℥viij. M. S. A sixth part to be taken three times a day.

"During an active practice of more than thirty-five years, I have met with several such cases, and in the times of depletion used to resort to the lancet with considerable success, but of late I have been in the habit of employing with benefit the following liniment: ℞. Liquoris ammoniæ fort, ʒiij.; tincturæ belladonnæ, tincturæ opii, aa. ʒiij.; liniment. sapon. ad, ℥iv. M. Relief is immediately experienced by a change of position from sitting to standing, and by leaning toward the left side, or lying on it.

"Small doses of hydrargyrum cum cretâ, combined with soda, and followed occasionally by more active aperients, and the internal remedies which I have seen of most avail. Externally, pledgets of lint soaked in laudanum seem to give most relief. I have never found it necessary to employ leeching."

Strychnia in Typhoid Fever.—Dr. F. R. Millard, of Beetown, Wisconsin, writes us: "I am not aware that strychinia, or nux vomica is used in typhoid fever; but having witnessed its curative action in dysentery, I was induced to give it a trial, and am satisfied that it is one of the best remedies we possess for supporting the patient and restraining the excessive action of the bowels. I seldom give more than one grain of extract nux vomica at a dose, repeated every four to eight hours, generally combined with opium. Either opium and ext. nux vomica aa.; or, opium one part, and ext. nux vomica two parts. I think that if my professional brethren will give it a trial, they will find it generally superior to quinine (in cases not decidedly complicated with malaria), not only in dysentery, but typhoid fever also."—*Medical and Surgical Reporter.*

Incontinence of Urine.—Dr. Holmes Coote, of St. Bartholomew's, recommends for incontinence of urine in children, one minim of creosote three times daily, "combined with asafœtida and rhubarb pill, of each two grains."

Apparent Death.—Dr. W. H. Lathrop, Professor of Diseases of the Brain and Mind in Detroit Medical College, (*Detroit Review of Medicine and Pharmacy*), in his paper on "Apparent Death," reports one of the most remarkable and best authenticated cases of trance on record, that of William (afterward Rev. Dr.) Tennent. While studying theology at Princeton, New Jersey, he had a fit of catalepsy, being in a very weak state from close application to books, without proper attention to physical exercise. After a short illness he was regarded as dead, and the day was fixed for the funeral. His physician, however, believed him to be alive, and succeeded in having the funeral postponed from day to day for three days. The brother of Mr. Tennent then insisted that it was folly to suppose that he could be alive, and ordered that at a certain hour the burial service should occur. When the appointed time arrived, and the company were assembled, the doctor stood over his patient and asked for still another hour. At the end of that time the patient suddenly uttered a groan, and soon after gave other signs of life. His recovery was slow, and at first was attended with a loss of memory. He commenced anew the study of Latin with as much difficulty as if he had had no previous acquaintance with the language. After a considerable time his memory suddenly returned. This case is fully authenticated. The witnesses were educated persons, and all the particulars have been carefully described. Such cases, therefore, are possible, though extremely rare.

In conclusion, Dr. Lathrop observes: 1. That trance is extremely rare. 2. That apparent life in those really dead is much more common. 3. That in doubtful cases we should wait for the appearance of decomposition before allowing the burial to take place.—*Medical News and Library.*

Delivering the Placenta.—Dr. S. P. Crawford (*Nashville Medical Journal*) remarks: "I have made it a point, for the last twenty years, to deliver the placenta at once. So soon as the child is taken away, I introduce my right hand partly into the womb, or until I can grasp the placenta; which is generally partly in the os, and with my other hand on the abdomen, and by gentle extracting force and manipulation, the womb contracts, and expels its contents at once."

Atropia Poisoning.—Thomas Opie, M.D., (*Physician and Surgeon*) by way of corroborating and giving prominence to the view that the physiological effects of opium are antagonistic to those of belladonna, reports a case of poisoning by atropia successfully treated by tincture of opium.—*The New York Medical Record.*

Effects of Compressed Air.—Dr. Etheridge, in a lecture published in the *Chicago Medical Journal*, gives some interesting details of experiments with compressed air. Patient is placed in an air-tight bell, and the air pumped in gradually till the necessary compression is obtained. Among the effects noticed are:

1. The respirations become slower, longer, and connected with a sensation of easiness.

2. There is an unusual completeness of the capillary circulation, and a decrease of the heart pulsations.

3. The power of the entire muscular system is increased.

4. Digestion is improved in every respect.

5. Urine is increased in quantity, sulphates are increased, and phosphates continually decrease till they disappear.

6. The weight is invariably increased by the prolonged use of the baths. In one case it is increased eight pounds in thirty-eight days. In another, ten pounds were gained in twenty-one days. From these facts it is apparent that nutrition is made more normal and better by an increased capillary movement. It has proved beneficial in catarrhal deafness, laryngitis, chronic bronchitis. In convalescence, in diseases of the lung—parenchyma, tuberculosis of lung, etc.—we judge that its effects are very similar to those of pure oxygen, and produced in the same manner.—*Detroit Review of Medicine and Pharmacy.*

The Influence of Bromide of Ammonium on Quinia.—H. C. Bell, M.D., of Colman, Missouri, (*Kansas City Medical Journal*) records his observations, with cases, on the corrective influence exerted by the bromide of ammonium on the action of quinia. By the use of the two agents in combination, he has succeeded in entirely preventing the cinchonism that usually follows the use of quinine in quantities sufficient to destroy the malarious influnce; and, in cases wherein it did not succeed, its influence was clearly seen, the intensity of the cerebral excitement being sensibly diminished. He has also found that the bromide of ammonium exerts less influence on those cases that had repeatedly taken quinine in quantities sufficient to produce severe, and often repeated, cinchonism; yet, even in such cases it has not proved entirely powerless. In no case has it apparently diminished the effect of the quinine; but, on the contrary, has seemed rather to serve as an auxiliary to its action, by holding in check the nervous system, equalizing the circulation, and thereby affording a more favorable condition of system for the action of the antiperiodic.

Dr. Hamilton's Means of Arresting Hemorrhage after Excision of Tonsils.—Cold applications to the neck. He prefers, when circumstances will permit, snow enveloped in a neck cloth.

Spasmodic Asthma.—Dr. Theodore C. Miller (*Journal of Materia Medica*) says: "I have found oxalate of cerium of great service in this affection; also the permanganate of potass. The latter remedy I have been in the habit of using as follows: ℞. Potass. permanganate, gr. ii.; aqua distill., ℥ i. M. One teaspoonful three times daily.

"The lactate of zinc has latterly grown into favor with me. In some obstinate cases in which I have employed this agent, it has given prompt relief. My prescription is: ℞. Zinc lactatis, gr. xx.; pulv. valerianæ, ext. valerianæ, aa. q. s. M. f. pil. No. x. One, morning and evening. I have employed lactate zinc in eclampsia of children, and regard it an invaluable medicine to combat this disease.

"*Faba St. Ignatii.*—Prof. Weber praises this agent in eclampsia of children, to control the epileptic attack resembling convulsions in children, particularly during dentition, and in anæmic children who have a highly nervous temperament and a weak constitution, he used it in the following form: ℞. Tinct. Faba St. Ignatii, gtts. 1–3; aqua distill., ℥ ii.; sirup altheæ, ℥ ii. M. A small teaspoonful every ½–1 hour.

"In my own practice the St. Ignatia bean in same form, has proven of much service in cholera infantum, diarrhœa of children and adults, and in prolapsus ani."

Uterine Hemorrhage.—Dr. G. C. Petzer (*Eclectic Medical Journal*) says: "When I tell you that cinnamon and erigeron are specifics in uterine hemorrhage, and that I know it by actual experience of several years, I don't tell you anything new, but I recall your attention to the fact, and confirm, so far as my evidence goes, what has been said of these articles by others. Let me say while speaking of these invaluable remedies that, in uterine hemorrhage, you can't have too much confidence in them. They are just what you want. Don't resort to ergot. Give oil erigeron gtts. x. every hour, and oftener if needs be, and between each dose give gtts. xx. tinct. oil cinnamon, made by adding oil cinnamon f℥ to alcohol f℥j. I use both remedies in every case, alternating. Don't know which does the most good, neither do I care much, just so I save my patient."

Reid's Cholera Sirup.—℞. Chloroform, ℥j.; gum camphor, ℥iij.; tinct. opium, Oj.; best French brandy, Oij.; tinct. cloves, ℥viij.; simple sirup, cong. ss. "As soon as bowel complaint makes its appearance, take a teaspoonful of the sirup and go to bed; repeat the dose every time the bowels are moved, increasing it if the first proves inefficient. Children should be dosed in proportion to age."

PREVENTION OF MAMMARY ABSCESS.—W. R. G. Samuel, in a communication to the *Lancet*, commending the use of olive oil as a local application to prevent mammary abscess, says:

"The mode I usually employ is to cut a piece of lint the size of each breast, allowing it to come well under the axilla, leaving a hole for the nipple to pass through for the infant to suck. Before applying the lint, saturate it well with the best olive oil and envelop the breast; next, cut a piece of oiled silk the shape and size of the lint, and cover this over the breast so as to prevent the oil from soiling the linen. In all cases let this be applied not later than the second day after the *accouchement*. As the lint becomes dry, moisten with more oil. Let this be applied until the patient is able to get about. I have tried it in many cases, several of which were primipara, and in no single instance has it failed. Besides, it not only prevents the obstruction of the milk-ducts, by its heating and emollient properties stimulating the capillaries around the base of the gland, but it also causes the milk to flow from the excretory ducts involuntarily (tubuli lactiferi), or, what is better understood in the phraseology of nurses and midwives, by causing the breasts to run. I am of opinion that if the oil is properly applied, and at the proper time, there no longer remains cause to fear the formation of mammary abscess."—*Nashville Journal of Medicine and Surgery.*

HEPATIC ABSCESS CURED BY INJECTIONS OF IODIZED IODIDE OF POTASSIUM.—A merchant, fifty-two years of age, was seized with deep lacerating pain in the right hypochondrium, followed, fifteen days afterward, with swelling in the iliac region of the same side. After two months of intense suffering the tumor opened spontaneously, discharging a large quantity of fetid pus. For three years this patient underwent different modes of treatment without any amelioration of the local difficulty. His constitution was greatly impaired when Dr. Goldsberry was called in. Dr. G. commenced by injecting into the fistulous sinuses tepid water twice a day; then a solution of four grains of iodine and eight of iodide of potassium in a pint of water. The strength of this solution was gradually increased as the patient was able to bear it. After making use of this treatment for one year, all flow of pus and bile had ceased, the fistulous tracts were entirely closed, and the general condition was very satisfactory. There was no relapse.—*La France Medicale.*—*Chicago Medical Examiner.*

FORMULA FOR CONSTITUTIONAL SYPHILIS.—℞. Iodide of sodium, ʒiij.; iodide of potassium, ʒij.; aqua lauro sassafras, ℥xivss.; syr. ferri protiodidi, ℥i.; extract corydalis formosa, ℥ss.; extract asclepias syriaca, ʒj. Mix. S. A tablespoonful before each meal.

INDUCTION OF PREMATURE LABOR.—Dr. Clement Godson, (*London Lancet*), gives the following method: "The means I advocate operates by surely and safely coaxing the uterus into an action which only differs from natural labor in being artificially initiated, and which is maintained and completed under all the conditions of labor spontaneously occurring at a corresponding stage of pregnancy. Each of the methods in general use is, according to my experience, more or less formidable, in virtue of the amount and the kind of the manipulation which it involves. Most of them are practiced in such a manner as to force on too hurriedly the uterine contractions; and that which consists in the evacuation of the liquor amnii stands self-condemned, as depriving the womb at the very outset of the all-important dilator provided by nature. My mode of procedure consists in insinuating, night and morning, between the cervix uteri and the membranes, sponge tents of gradually increasing size, the first and each succeeding one being as large as the parts will admit. On removing each tent, and before replacing it by another, a warm douche, containing Condy's fluid, is administered. I have found the use of one, two and three tents to be sufficient, and have never had occasion to employ more than four."

IODINE.—Inquiry is often made regarding the composition of the "iodine caustic" and the "compound tincture of iodine" of Churchill. By the kindness of Dr. William Neergaard, we are able to give the formulæ as follows: Churchill's iodine caustic is composed of "iodine, one drachm; iodide of potassium, two drachms; water, half a fluid ounce; mix." This mixture will be seen to be more than five times the strength of liq. iodinii co. of the U. S. Pharmacopœia, which it otherwise resembles. Churchill's compound tincture of iodine is: "Pure iodine, two and a half ounces; iodide of potassium, half an ounce; alcohol, four fluid ounces; rectified spirit of wine, twelve fluid ounces; mix them." Each fluid ounce of this tincture contains seventy-five grains of iodine, five times as much as the tincture bearing the same name in the U. S. Pharmacopœia.—*New York Medical Journal.*

COMPOUND ARSENICAL PAPER.—℞. Belladonna leaves, grs. xcvj.; hyoscyamus leaves, stramonium leaves, aa. grs. xiviij.; ext. opium, grs. iv.; tobacco, grs. lxxx. Add potass. nit., grs. cxx.; potass. arsenit., grs. cccxx. Take thick bibulous paper; soak it in this solution, and allow it to dry. When set on fire and the flame extinguished, this paper burns slowly without flame, and emits a dense smoke which may be inhaled for the relief of asthma, often with very marked benefit. It is also useful in chronic bronchitis,

Abortive Treatment of Boils and Felons (*Gaz. Med. Ital.*, November).—We find quoted from the *Giorn. dell Accad. Med. de Torino* the following method of treating boils and felons, which Dr. Simon regards as almost infallible: "Wherever the boil may be, and of whatever size, so long as suppuration has not commenced, rub it gently with the finger wet with camphorated alcohol, pressing especially on its centre. Do this half a minute at a time for seven or eight times, and then cover the part with camphorated olive oil. If one operation does not produce resolution, repeat it at intervals of six hours. A felon may be bathed ten minutes in camphorated alcohol, then dried and covered with the oil. The writer has never known a felon fail to succumb to three of these operations.

Pain in the Bladder or Penis.—A patient complains of pain in the region of the bladder or perineum. There is almost certainly chronic cystitis. Ask whether he feels the pain before, during, or after passing urine. If the pain is before, it is because the mucous membrane is becoming uneasy in consequence of distension. If the pain is during and after passing water, and in the end of the penis, he is likely to have stone; and especially also if the pain is increased by exercise. It is almost pathognomonic of stone to have the pain in the tip of the penis. Chronic prostatitis simulates stone more than any other disease. In both, the pain is at the tip of the penis.—*Braithwaite.*

Hyoscyamia.—1. Hyoscyamia exerts a favorable action in spasmodic and convulsive neuroses. It cured mercurial trembling in cases where every other remedy had failed. It produced a notable improvement in senile tremor and paralysis agitans. 2. Hyoscyamia should be given at first in weak doses (one thirty-two grain daily), either in pills or by hypodermic injection. The dose may be gradually increased to five or six times this quantity. 3. The remedy must be continued, even if there are slight symptoms of poisoning, such as dryness of the throat and dilated pupils. But if the symptoms become grave, it must be suspended, and the symptoms being temporary, will disappear in a few hours.

Impotency.—Prof. D. Hayes Agnew, of Philadelphia, (*Medical and Surgical Reporter*), in a clinical lecture on impotency, after stating that the treatment must be largely moral, and urging the abandonment of onanism or excessive venery, says that there is no better internal treatment than phosphorus and strychnia—1-50 grain of the former, and 1-30 of the latter—made into a pill, and administered three times daily.—*Clinic.*

Treatment of Anthrax by Subcutaneous Aspiration (*Le Mouvement Médical*, January, 1873).—We believe that the most effective treatment of anthrax is by crucial incision. Anthrax may also be opened subcutaneously (J. Guérin); an operation which has the advantage of being less painful than the preceding. For timid patients the following treatment may be of service: The canula of a hypodermic syringe is introduced into the center of the tumor, and pus, if any be present, will rise on drawing the piston. The syringe is detached and emptied, leaving the canula in the tumor; it is then readjusted to the canula, and the piston drawn so long as pus continues to rise. When the pus is completely evacuated, the canula is taken out, and the following preparation is applied to the tumor: ℞. Collodion, four grammes; castor oil, gtts. xx.; phenic acid, cgr. 25; tannin, 1 to 30. The applications are to be made until a sufficiently thick layer is obtained.

Iodine in Chronic Diarrhœa.—Five drops compound solution of iodine in half a teaspoonful of water, four times a day, is recommended by a writer in the *Buffalo Medical Journal.* The external application of iodine mixed with olive oil is reported to have been very useful in obstinate cases. Dr. Hartshorne, of Philadelphia, recommends an enema of twenty grains sulphate of zinc and a grain or two of opium, in four ounces of water, repeated several times.—*Pacific Medical and Surgical Journal.*

Nitric Acid for Hemorrhoids.—Billroth, of Vienna, has lately revived an old treatment of hemorrhoids connected with prolapsus, consisting simply of the application of nitric acid by a hair pencil. We have great confidence in the remedy, especially in recent cases without constitutional complications. A very few applications will sometimes cure promptly and with trifling pain. It should always be tried before resorting to surgical extirpation. *Pacific Medical and Surgical Journal.*

Belladonna in Intestinal Invagination and Hernia.—Dr. Gallicier thus sums up an interesting paper which he has published on the above in *La France Medicale:* "Belladonna is, therefore, the special medicament of intestinal invagination, strangulated or not, as also of strangulated hernia. It acts both on the spasmodic element and the inflammatory element. In both cases, employed *intus et extra*, its first effect is to alleviate the intensity of pain, and to diminish or arrest the vomiting."

An Application to Corns.—Castor oil applied to the corn after paring closely each night before going to bed. It softens the corn, and it becomes as the other flesh. It will cure every time,

THE NATURAL CURE OF DISEASE.—Samuel G. Armor, M.D., of Brooklyn, New York, (*New York Medical Journal*, January, 1873), has a lecture on this subject, being replete in sound sense, and an introductory to the discussion of therapeutics. He emphasizes the following rules of practice: 1. Never administer a drug of any potency without a definite purpose—that is, without a clear indication—for drugs never occupy neutral ground. 2. Never use more medicine than is requisite to produce the effect which is intended, and continue it no longer than is absolutely necessary. In closing, he would enforce these truisms: It is the duty of the physician to restore health by the simplest means in his power. To know the natural cure of disease is more than half of therapeutics.—*New York Medical Record.*

OPERATION FOR FISTULA IN ANO.—Dr. W. S. Parker (*Clinic*) gives the following operation: "The bowels having been first well cleared, I insert a rectal speculum with a fenestra of sufficient length into the rectum, and then introduce through the fistula a grooved director to within the speculum. The intervening tissues are then conveniently divided, the cut being simply a smooth one, and devoid of any tearing or crushing, as must occur in the method where the knife is thrust into a stick of wood within the rectum, and the two withdrawn simultaneously. I think the operation much superior also to the plan of passing the director through the fistula and dragging it out and laying it across the buttocks. The manner of healing from the bottom of the wound remains the same."

SALVE FOR THE LIPS, ETC.—The following is used for excoriations, warts and cracks, superficial ulcerations of the lips, mucous membrane of the cheeks, red parts of margin of nose, and other parts of the skin which are normally red: ℞. Ammoniated mercury, gr. vj.; carmine, gr. i.; soft cerate, ʒ ij. Mix exactly. The parts should be dried before the salve is applied.

FOR EXCESSIVE PERSPIRATION OF HANDS OR FEET.—A German pharmaceutical journal recommends the following: ℞. Carbolic acid, one part; burnt alum, four parts; starch, two hundred parts; French chalk, fifty parts; oil of lemon, two parts. Make a fine powder, to be applied to the hands and feet, or to be sprinkled inside of the gloves or stockings.

RANULA CURED BY GALVANO-ACUPUNCTURE (*Nuou Ligur. Med.*, October 26).—Dr. Giovanni Gasparini reports several cases of ranula cured in this way. Two needles are just as effective as more, and the electric current need not and ought not to be a strong one.

SQUIRE'S VERTEBRATED PROSTATIC CATHETER.—This instrument is like an ordinary silver catheter in appearance, but the last three or four inches is composed of a series of perfect joints, so as to allow of a large amount of flexibility. Thus, the instrument is sufficiently flexible to accommodate itself to any curves of the prostatic portion of the urethra, but it cannot double upon itself as a flexible gum-elastic or caoutchouc catheter sometimes does. At the same time, it possesses longitudinal stability, that the propulsion of the hand may be transmitted without loss to the beak. This instrument has been very much approved of in New York.—*Braithwaite's Retrospect.*

BROMIDE OF POTASSIUM IN EPILEPSY.—M. Voisin, in a full report on this drug in the *Archives Generales de Medicine,* says: "Its efficacy in epilepsy is incontestable, even where a great number of attacks have occurred (four thousand), or when the disease is of long duration (fifteen years). Cure is interfered with by organic causes, hereditary tuberculosis and alcoholism, malformation of the brain, onanism, plastic effusions, sclerosis, clots, softening, conditions often causing loss of special sense or motor power. Menstrual epilepsy is less favorably affected by the bromide. On this subject M. Voisin gives ample details."—*Med. and Surg. Rep.*

HOW TO DISSOLVE ALUM IN BREAD.—One drop of the alcoholic extract of logwood let fall upon pure bread or pure flour, gives a color brownish-yellow. If the flour contain alum in the proportion of one or two per cent., the color is gray or grayish-violet; with half per cent., the spot is yellowish-red, with a border of bluish-gray, and little blue points may be discovered in the disc with a lens; with a quarter per cent., the bluish border is no longer visible, but the points may still be detected. This is the limit of the reaction.—*Mouvement Médicale.*

VICARIOUS MENSTRUATION.—M. Gayet, of Hôtel Dieu, records a curious case of vicarious menstruation in a young girl of seventeen, who had never regularly menstruated, yet every month she would lose for two or three days a considerable amount of blood from a fissure around either of the nipples. As soon as menstruation *per vias naturales* established itself, this hemorrhage ceased.

IMMEDIATE REMEDY FOR HEMOPTYSIS.—A saturated solution of gallic acid, inhaled by means of an atomizer, is recommended by Dr. Holden, in the *New York Medical Record,* as capable of arresting pulmonary hemorrhage with great promptness, even while the flow of blood is profuse. In urgent cases he mixes the acid with cold water and uses it without waiting for solution to be perfected.

